# Dual-function DEFENSIN 8 mediates phloem cadmium unloading and accumulation in rice grains

**DOI:** 10.1093/plphys/kiac423

**Published:** 2022-09-10

**Authors:** Tian-Yu Gu, Zi-Ai Qi, Si-Ying Chen, Jing Yan, Zi-Jun Fang, Jun-Min Wang, Ji-Ming Gong

**Affiliations:** National Key Laboratory of Plant Molecular Genetics, Center for Excellence in Molecular Plant Sciences, Shanghai Institute of Plant Physiology and Ecology, Chinese Academy of Sciences, Shanghai 200032, China; School of Life Science and Technology, ShanghaiTech University, Shanghai 201210, China; University of the Chinese Academy of Sciences, Beijing 100049, China; National Key Laboratory of Plant Molecular Genetics, Center for Excellence in Molecular Plant Sciences, Shanghai Institute of Plant Physiology and Ecology, Chinese Academy of Sciences, Shanghai 200032, China; University of the Chinese Academy of Sciences, Beijing 100049, China; National Key Laboratory of Plant Molecular Genetics, Center for Excellence in Molecular Plant Sciences, Shanghai Institute of Plant Physiology and Ecology, Chinese Academy of Sciences, Shanghai 200032, China; University of the Chinese Academy of Sciences, Beijing 100049, China; National Key Laboratory of Plant Molecular Genetics, Center for Excellence in Molecular Plant Sciences, Shanghai Institute of Plant Physiology and Ecology, Chinese Academy of Sciences, Shanghai 200032, China; State Key Laboratory of Crop Stress Adaptation and Improvement, School of Life Sciences, Henan University, Kaifeng 475004, China; National Key Laboratory of Plant Molecular Genetics, Center for Excellence in Molecular Plant Sciences, Shanghai Institute of Plant Physiology and Ecology, Chinese Academy of Sciences, Shanghai 200032, China; Institute of Crops and Nuclear Technology Utilization, Zhejiang Academy of Agricultural Sciences, Hangzhou 310021, China; National Key Laboratory of Plant Molecular Genetics, Center for Excellence in Molecular Plant Sciences, Shanghai Institute of Plant Physiology and Ecology, Chinese Academy of Sciences, Shanghai 200032, China; School of Life Science and Technology, ShanghaiTech University, Shanghai 201210, China; University of the Chinese Academy of Sciences, Beijing 100049, China

## Abstract

Grain cadmium (Cd) is translocated from source to sink tissues exclusively via phloem, though the phloem Cd unloading transporter has not been identified yet. Here, we isolated and functionally characterized a defensin-like gene *DEFENSIN 8* (*DEF8*) highly expressed in rice (*Oryza sativa*) grains and induced by Cd exposure in seedling roots. Histochemical analysis and subcellular localization detected *DEF8* expression preferentially in pericycle cells and phloem of seedling roots, as well as in phloem of grain vasculatures. Further analysis demonstrated that DEF8 is secreted into extracellular spaces possibly by vesicle trafficking. DEF8 bound to Cd in vitro, and Cd efflux from protoplasts as well as loading into xylem vessels decreased in the *def8* mutant seedlings compared with the wild type. At maturity, significantly less Cd accumulation was observed in the mutant grains. These results suggest that DEF8 is a dual function protein that facilitates Cd loading into xylem and unloading from phloem, thus mediating Cd translocation from roots to shoots and further allocation to grains, representing a phloem Cd unloading regulator. Moreover, essential mineral nutrient accumulation as well as important agronomic traits were not affected in the *def8* mutants, suggesting *DEF8* is an ideal target for breeding low grain Cd rice.

## Teaser

The defensin family member DEFENSIN 8 (DEF8) mediates both xylem cadmium (Cd) loading and phloem Cd unloading via the chelation and secretion mechanism in rice.

## Introduction

Rice (*Oryza sativa*) is a major staple food and a substantial part of the human diet (Huang et al., 2012). Cadmium (Cd) is a non-essential element that can accumulate in rice grown in polluted soils, and is toxic to humans. Cd also affects cell division, metabolism, and growth of plants, leading to a severe decline of yield and quality (Clemens and Ma, 2016). Therefore, it is of great importance and economic value to control Cd accumulation in rice grains. Several essential steps could be considered to control Cd accumulation in rice grains, which include Cd uptake into roots, root-to-shoot transport, and allocation between stems and panicles, and this whole process is tightly controlled by various transporters.

Natural resistance-associated macrophage protein 5 (OsNRAMP5) was identified as the major transporter controlling Cd uptake into rice roots (Sasaki et al., 2012), and it is polarly localized to the outer side of the plasma membrane of the root exodermis and endodermis. Functional disruption of OsNRAMP5 significantly decreased Cd accumulation, as well as the essential ion Mn which resulted in substantial loss in biomass and grain yield (Sasaki et al., 2012). Exogenous Mn addition restored the growth defect in the *osnrampr5* mutant plants (Wang et al., 2019). *OsCd1*, a member of the major facilitator superfamily, is another quantitative trait locus (QTL) gene contributing to Cd uptake into roots, and the japonica allele *OsCd1*^V449^ significantly reduced Cd uptake and accumulation in rice grains (Yan et al., 2019). Other transporters are also involved in the uptake process, among which OsNRAMP1 was identified to mediate Cd and Mn uptake (Chang et al., 2020), and the Fe transporter IRON-REGULATED TRANSPORTER1 (OsIRT1) has been proposed to promote Cd uptake under iron deficiency condition (Lee and An, 2009).

In terms of Cd allocation from root to shoot or shoot to panicle, OsHMA3 (Heavy-Metal ATPase3) is believed to be a major player. It is localized to the tonoplast of rice roots, and serves as a “firewall” preventing long-distance Cd translocation by sequestering Cd into root vacuoles (Ueno et al., 2010; Miyadate et al., 2011; Peng and Gong, 2014). In the high Cd-accumulation cultivar Anjana Dhan, a loss-of-function allele of *OsHMA3* resulted in less Cd sequestration into vacuoles and hence more translocation to aerial parts, while overexpression of a functional OsHMA3 essentially decreased Cd accumulation in rice grains (Ueno et al., 2010). A recent study further revealed that sequence variation in the *OsHMA3* promoter determines the divergent grain Cd accumulation between *Indica* and *Japonica* rice by modulating *OsHMA3* expression (Liu et al., 2020). OsHMA2 is another important regulator in mediating long-distance Cd translocation, which functions to directly load Cd as well as Zn from pericycle cells into xylem vessels (Takahashi et al., 2012). Later research further revealed that OsHMA2 also mediates distribution of Zn and Cd to panicles via phloem loading (Takahashi et al., 2012; Yamaji et al., 2013). Low-affinity cation transporter (OsLCT1) might represent the only transporter identified to date to specifically mediate phloem Cd loading, as knockdown of this gene reduced phloem-mediated Cd transport but not xylem-mediated Cd transport, nor did the mutation affect other metal accumulation in rice grains (Uraguchi et al., 2011). To date, no phloem Cd unloading transporter has been identified.

Recent studies identified a mechanism of Cd long distance transport mediated by defensin-like proteins. Plant defensins are small cysteine-rich proteins, which consist of a secretion signal peptide (SSP) and a cysteine-rich domain (regularly 8 Cys residues) that binds to metals (Mirouze et al., 2006; Shahzad et al., 2013). Cd accumulation in leaf 1 (*CAL1*) is a quantitative trait locus gene encoding a defensin-like protein, chelating Cd in the cytosol and hence driving Cd secretion from cytosol to extracellular spaces in rice (Luo et al., 2018). In this way, it lowers the cytosolic Cd concentration meanwhile promotes Cd long distance transport and specific accumulation in shoots (Luo et al., 2018). CAL2, a homolog of CAL1, works similarly to CAL1 but expresses preferentially in root tips, however, it significantly increased grain Cd via an unknown mechanism when overexpressed (Luo et al., 2020). These observations suggest that defensin family members might play crucial roles in Cd transport and distribution, and possibly represent ideal breeding targets to specifically modulate Cd accumulation in rice grains.

In the current study, we report the functional characterization of defensin 8 (*DEF8*), a defensin family gene that is highly expressed in rice grains. Our results demonstrate that DEF8 has dual function in mediating both root-to-shoot long distance Cd transport and phloem Cd transport to grains, thus it might represent a phloem Cd unloading “transchelator”: translocating Cd across membranes via chelation and secretion.

## Results

### *DEF8* is highly expressed in rice grains

**Figure 1:**
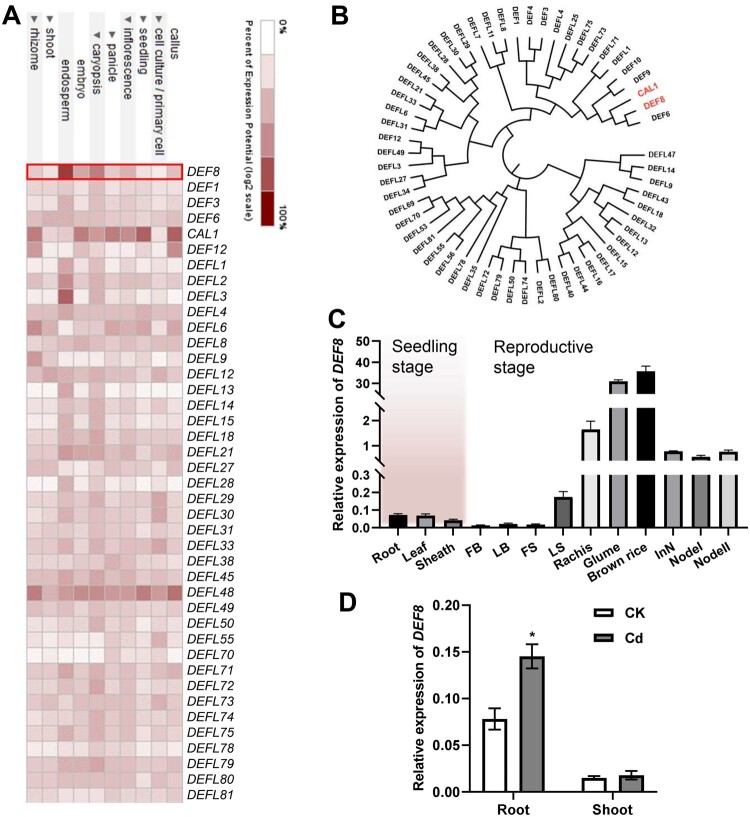
The defensin family member *DEF8* is highly expressed in rice grains. A, Expression of the defensin family members in different anatomical parts. Data were obtained and profiled according to Genevestigator. The darkest burgundy color represents the “maximum” level of expression for a given probe across all measurements available in the database. B, Phylogenetic tree for DEF family members in rice was constructed using MEGA7 based on the multiple sequence alignments of defensin proteins from EMBL-EBI database, using neighbor-joining method of the ClustalX program. *CAL1* and *DEF8* were highlighted in red. C, Expression pattern of *DEF8* in various rice tissues. Root, leaf, and sheath were harvested from 2-week-old seedlings. LB (leaf blade), FB (flag leaf blade), FS (flag leaf sheath), LS (leaf sheath), rachis, glume, brown rice, InN (internode), Node I, and Node II were harvested during the reproductive stage. Values are mean ± sd and normalized to *Actin1*, *n* = 3. D, *DEF8* expression in 2-week-old seedlings treated with 0 µM (CK) or 10 μM (Cd) Cd for another 7 days. *Actin1* was used as an internal control. Values are mean ± sd, and significance was determined by ANOVA test (*P* < 0.05), *n* = 4.

To identify key defensin genes responsible for grain Cd transport, we profiled the digital expression of all the defensin family genes in rice through Genevestigator (https://genevestigator.com). *DEF8* was selected because it is expressed at much higher levels in endosperm than other members (Figure 1A), and the expression is steadily elevated during grain ripening (Supplemental Figure S1). Phylogenetic analysis showed that DEF8 shares the highest identity with CAL1 (Figure 1B). The full-length precursor of DEF8 (_f_DEF8) contains a putative SSP and a cysteine-rich (eight cysteine residues) domain which represents the truncated mature DEF8 (_t_DEF8) after removal of the SSP (Supplemental Figure S2A). Further reverse transcription quantitative PCR (RT-qPCR) assay indicated that *DEF8* was ubiquitously expressed at relatively low levels in seedlings, but at much higher levels especially in rachis, glume, and brown rice during the reproductive stage (Figure 1C). *DEF8* expression upon Cd treatment was significantly upregulated in seedling roots but not in shoots (Figure 1D). These results suggest that DEF8 might function in a similar way as CAL1 does, and mediate Cd and/or other metals’ loading to rice grains.

### DEF8 is secreted to extracellular spaces possibly via vesicle trafficking

**Figure 2:**
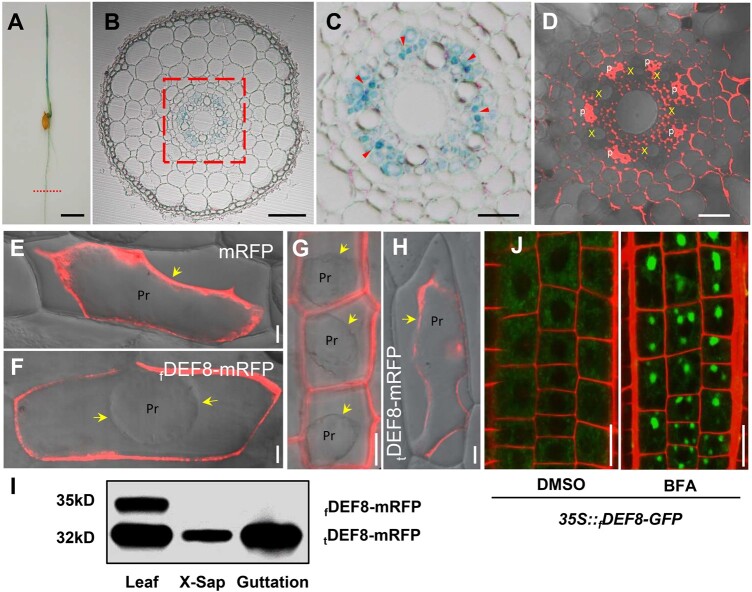
Histochemical analysis and localization of DEF8. A–C, GUS expression driven by the *DEF8* promoter at the seedling stage. Histochemical assay of 7-day-old rice seedlings (A), root cross section (B) and zoom in from (B) in dashed box (C). Arrow heads indicate phloem. Bar = 1 cm in (A), 50 µm in (B), and 10 µm in (C). D, Root cross section of rice seedlings harboring the construct *proDEF8::_f_DEF8-mRFP*. P, phloem; X, xylem. Bar = 25 μm. E–H, Subcellular localization of DEF8. Onion epidermal cells were transiently transformed with *mRFP* (E), *35S::_f_DEF8-mRFP* (_f_DEF8-mRFP, F), and *35S::_t_DEF8-mRFP* (_t_DEF8-mRFP, H), and incubated in 30% sucrose to induce plasmolysis before confocal microscopy imaging, and protoplasts (Pro) are indicated by arrows. G, DEF8 localization in transgenic rice harboring *proDEF8::_f_DEF8-mRFP*. Epidermal cells of leaf sheath were incubated in 30% sucrose to induce plasmolysis. I, Detection of DEF8 by Western blotting. Rice seedlings from (G) were grown for 2 weeks in hydroponics. Proteins were extracted from leaf, xylem sap (X-Sap), or guttation fluid, respectively, and subjected to Western blotting assay using an anti-RFP antibody. J, Effect of BFA on DEF8 localization in root. Four-day-old transgenic Col-0 expressing *35S::_f_DEF8-GFP* was treated with 50 µM BFA for 30 min before imaging. DMSO-treated root cells were used as control.

To elucidate the function of DEF8, we first performed histochemical analysis, and the β-Glucuronidase (GUS) activity was detected in both roots and shoots of rice seedlings (Figure 2A). Further cross-section imaging indicated that the expression appeared to be located in pericycle cells and phloem within vasculatures (Figure 2, B and C), indicating a possible role in long-distance transport. A subcellular localization assay showed that DEF8 was detected in both phloem and extracellular compartments in root vasculature (Figure 2D). Further imaging of peeled cells showed that DEF8 is exclusively in walls of both the onion epidermal cells (Figure 2, E and F) and rice sheath cells (Figure 2G) that were transiently or stably transformed with the construct *_f_DEF8-mRFP*. However, when the SSP sequence was removed (*_t_DEF8-mRFP*, Supplemental Figure S2A), DEF8 was retained within the cell (Figure 2H), indicating that the SSP is essential to DEF8 efflux. Western blot detected both _f_DEF8-mRFP and _t_DEF8-mRFP in transgenic rice leaves, but only the mature _t_DEF8-mRFP was detected in xylem sap and guttation fluid (Figure 2I). These results suggest that DEF8 is secreted from cytosol into apoplastic spaces. Moreover, when treated with the vesicle transport inhibitor brefeldin A (BFA), vesicle bodies of DEF8 were formed in *Arabidopsis thaliana* roots transformed with *35S::_f_DEF8-GFP* (Figure 2J), suggesting that DEF8 is secreted possibly through vesicle trafficking pathway.

### Cd efflux via DEF8 secretion

**Figure 3:**
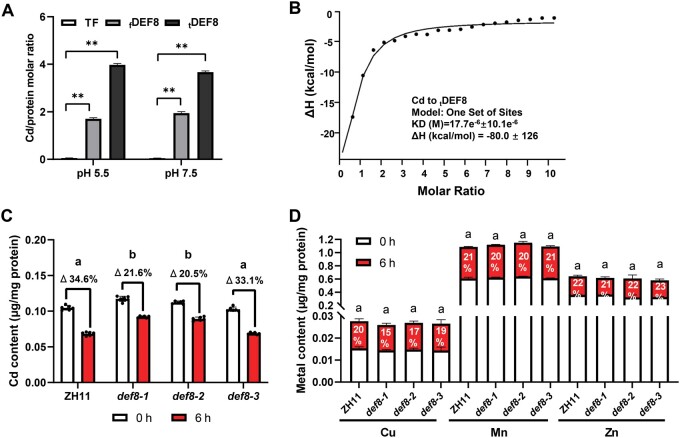
DEF8 mediates Cd efflux. A and B, Metal binding assay. Recombinant proteins TF-_f_DEF8 or TF-_t_DEF8 were purified from *E. coli* cells and incubated with 100 μM CdCl_2_ at pH 5.5 and 7.5 for 1 h before ICP-MS analysis to determine the stoichiometry of DEF8:Cd (A), and binding affinity of the mature _t_DEF8 to Cd was determined by ITC at pH 7.5 (B). TF represents the *E. coli* trigger factor protein fused to the N-terminus of target proteins. C and D, Cd efflux in protoplasts. Rice seedlings of the wild-type (ZH11) and *def8* mutants (*def8-1, def8-2, def8-3*) were grown for 14 days in hydroponics supplemented with 5 µM Cd, and leaf sheath was sampled to isolate protoplasts, which were then cultivated in W5 buffer for 6 h before determining the contents of Cd (C) or other metals as indicated (D). Numbers above the bars represent Cd decrease rate at 6 h compared with 0 h. Values are mean ± sd, and significance was determined by Student’s *t* test in (A) and ANOVA test (*P* < 0.05) in (C and D), *n* = 6. Asterisks indicate difference at *P* < 0.01 (**).

Using the recombinant proteins _f_DEF8 and _t_DEF8 ectopically expressed in *Escherchia coli*, we demonstrated that both the full-length and signal peptide-truncated DEF8 showed Cd binding activity at both pH 5.5 and 7.5, and the mature _t_DEF8 seemed to be more efficient (Figure 3A). Further isothermal titration calorimetry (ITC) analysis showed that DEF8 bound Cd (Figure 3B), as well as Cu but not Mn (Supplemental Figure S2, D and E). Considering the secretion of DEF8 to extracellular spaces, these results suggest that DEF8 might mediate Cd efflux via chelation and secretion. To test this postulation, we then generated rice mutant lines using CRISPR/Cas9 (Supplemental Figure S2B), and *def8-1* and *def8-2* were identified as loss-of-function mutants, while *def8-3* was used as a wild-type control, because the 3 bp deletion that occurred in the signal peptide region of *def8-3* did not affect its maturation and secretion (Supplemental Figure S2C). Protoplasts were then extracted from these plants grown with 5 μM Cd, and less Cd decrease was observed in *def8-1* and *def8-2* protoplasts than those of the wild-type control ZH11 and *def8-3* after 6 h incubation (Figure 3C). No significant change was observed for Cu, Mn, and Zn (Figure 3D). It is worth mentioning that leaf sheath was used to prepare protoplasts, mainly because it is composed of many aerenchyma without any living cells and lots of vascular bundles. Taken together (Figure 3, A–C and Supplemental Figure S2, B and C), our data suggest that DEF8 might specifically bind Cd in vivo and facilitate its secretion from cytosol to extracellular space, likely through vesicle trafficking. Noting that although DEF8 showed in vitro Cu binding (Supplemental Figure S2D), it did not essentially affect Cu accumulation in the *def8* mutant (Supplemental Figure S2, F and G) despite the tendency for a possible slower decrease in isolated protoplasts (Figure 3D), implying that DEF8 may be involved in Cu homeostasis, but the regulation would at least be cell-type or condition specific.

### DEF8 mediates Cd long-distance transport in rice seedling

**Figure 4:**
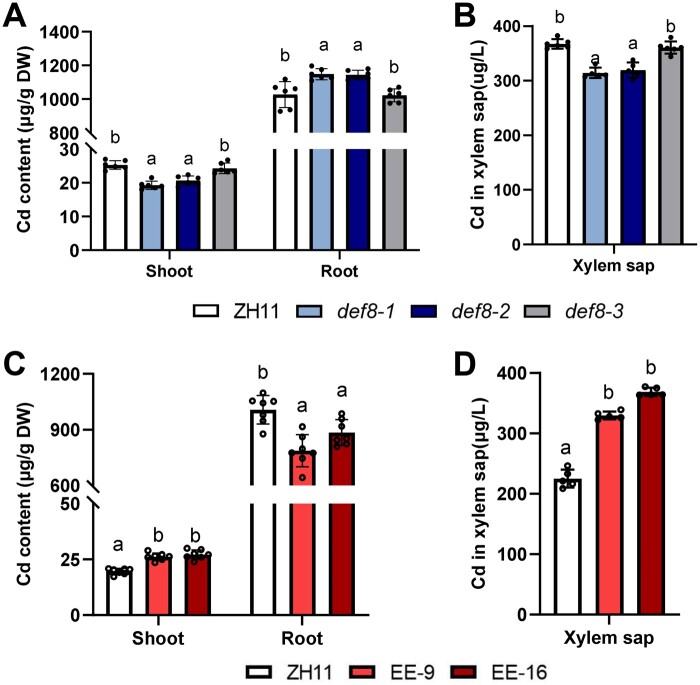
DEF8 mediates long-distance Cd transport in rice seedlings. A–D, Two-week-old seedlings were treated with 10 µM Cd for 7 days, then Cd contents in samples as indicated were determined for the mutants (A and B) or the DEF8-EE lines EE-9 and EE-16 (C and D). Values are mean ± sd, *n* = 5–7. Significant differences were determined by ANOVA test (*P* < 0.05).

Given that DEF8 facilitates Cd efflux to extracellular compartments and was detected in xylem and guttation fluid (Figures 2I and 3C), we wondered if DEF8 mediates long-distance Cd transport. To answer this question, we generated the transgenic plants elevated expression (EE)-9/EE-16, in which the construct *proDEF8::_f_DEF8-mRFP* was transformed and the *DEF8* promoter drove substantially EE of *DEF8* (Supplemental Figure S2H). Compared with the wild-type control ZH11 and *def8-3*, Cd concentration decreased in mutant shoots, xylem, and guttation fluid, and increased in their roots (Figure 4, A and B and Supplemental Figure S3A). No uptake difference was observed (Supplemental Figure S3, B and C). Consistently, an opposite Cd accumulation pattern was observed in the EE lines EE-9 and EE-16 (Figure 4, C and D). These results together suggest that DEF8 mediates Cd long-distance transport from root-to-shoot through xylem loading, and possibly also partially though reduced vacuolar Cd sequestration due to DEF8 binding. Further analysis showed that neither the seed germination rate (Supplemental Figure S4, A and B) nor the Cd sensitivity in terms of shoot/root length (Supplemental Figure S4, C and D) was affected despite the expression level of *DEF8* in rice.

### Phloem Cd transport via DEF8 in rice grains

**Figure 5:**
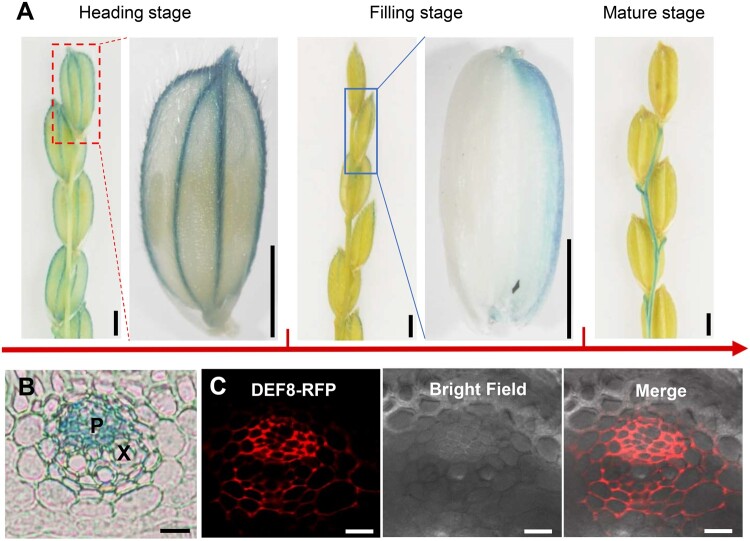
Characterization of DEF8 at the reproductive stage. A, Spatio-temporal expression of *DEF8* during reproductive growth. Histochemical assay of panicles from a transgenic plant harboring *proDEF8::GUS* at heading (left), filling (middle), and mature (right) stage. The part in the dashed box was enlarged to show staining of glume vasculature. The grain outlined in blue was shelled to brown rice. B, Cross-section of glume at heading stage. P, phloem; X, xylem. Bar = 10 µm. C, Imaging the cross-sectioned glume of transgenic plants harboring the construct *proDEF8::_f_DEF8-mRFP/pCAMBIA1300*. Bar = 10 µm.

**Figure 6:**
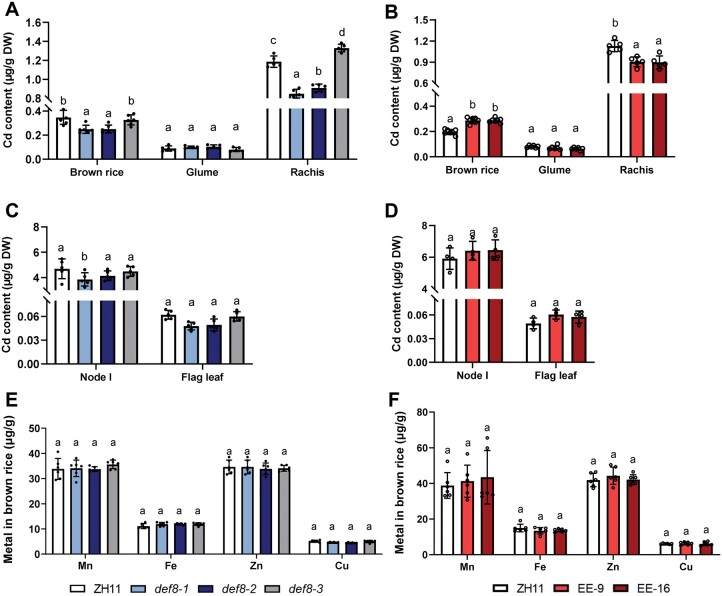
DEF8 mediates Cd distribution to rice grains. A–D, Cd contents in brown rice, glume, and rachis of *def8* (A) and transgenic plants with EE of *DEF8* (B), or in node I and flag leaves of *def8* (C) and transgenic plants (D). E and F, Essential metal accumulation in brown rice from plants in (A–D). Rice plants were grown in a Cd polluted paddy field until harvest, Values are mean ± sd, *n* = 5–6 in (A and B), 4–7 in (C and D), and 6 in (E and F). Significant differences were determined by ANOVA test (*P* < 0.05).

Given that *DEF8* showed much higher expression in ripening grain and adjoining tissues than in others (Figure 1C), we performed further histochemical analysis and found that *DEF8* expressed exclusively in vascular bundles of the glume at the heading stage, which became weaker at the filling stage and strong expression was instead observed in vascular bundles of the endosperm (Figure 5A, left and middle panels). At the maturation stage, *DEF8* expression was detected only in rachis (Figure 5A, right panel), possibly because silicon accumulated on the mature glume prevented GUS staining. Cross-section analysis showed that *DEF8* expressed in the phloem of the glume (Figure 5B), consistent with the subcellular localization of the DEF8-mRFP fusion protein (Figure 5C). These results suggest that DEF8 might function to unload Cd from phloem to the surrounding apoplastic compartments. Consistent with this hypothesis, Cd accumulation decreased in brown rice and rachis of the mutant compared with those of the wild-type control ZH11 and *def8-3* (Figure 6A). In EE-9/EE-6 with much higher *DEF8* expression, however, more Cd was detected in brown rice but not in rachis (Figure 6B). In lower tissues such as Node I and flag leaves, comparable accumulation was observed between the mutant and the wild type (Figure 6C), or between the EE transgenic plants and the corresponding wild-type control (Figure 6D). These results suggest that in addition to its role in long-distance Cd transport from root to shoot at the seeding stage, DEF8 also functions in Cd unloading from phloem to rice grains during grain filling.

Considering that DEF8 did show Cu binding activity (Supplemental Figure S2D), we then determined if DEF8 affects other metal accumulation, and the results showed that both in the mutant or EE transgenic plants, no significant difference was observed compared with the wild-type control (Figure 6, E and F), suggesting that DEF8 might specifically mediate Cd translocation through either xylem or phloem dependent on different development stages. Further field experiment indicated that important agronomic traits including plant height, tiller number, 1,000-grain weight, and seed setting rate were comparable between the wild-type and the mutant or EE transgenic plants (Supplemental Figure S5, A–D). Even when grown in a Cd-polluted field, comparable traits were also observed between those plants (Supplemental Figure S5, E and F). These results together suggest that DEF8 represents an ideal breeding target to modulate Cd accumulation in rice grains.

### Ectopic expression of *DEF8* increased Cd tolerance and translocation in *Arabidopsis*

**Figure 7:**
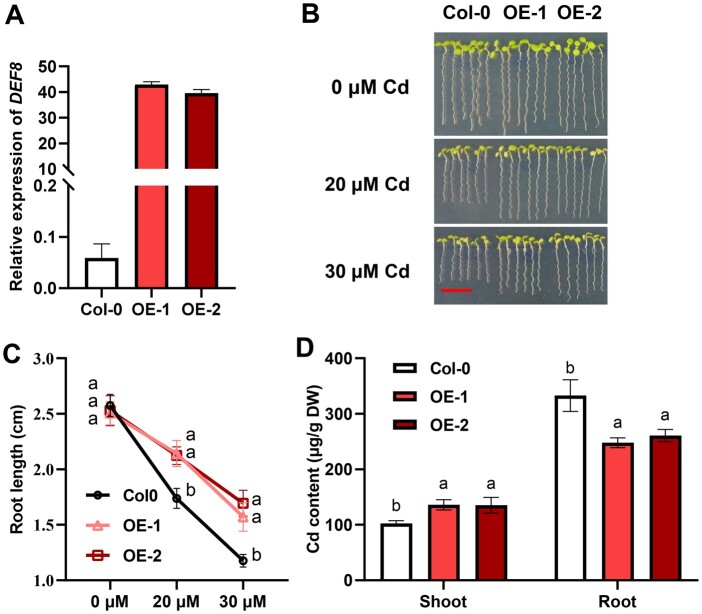
Ectopic expression of *DEF8* in *Arabidopsis*. A, *DEF8* expression in transgenic *Arabidopsis* plants. *Arabidopsis* plants harboring *35S::DEF8-GFP* (OE-1, OE-2) were sampled to determine the relative expression of *DEF8*. Values are mean ± sd, *n* = 3. *Actin1* was used as an internal control. B and C, OE-1 and OE-2 are more tolerant to Cd. Seedlings were grown on 1/2× MS medium with 0 µM, 20 µM, or 30 µM Cd for 7 days (B), and root length was measured (C). D, Cd contents in shoot and root*.* Four-week-old plants grown in hydroponics were treated with 10 µM Cd for 3 days before sampling for ICP-MS determination of Cd contents. Bar = 1 cm in (B). Values are mean ± sd, *n* = 12 in (C), 5–8 in (D). Significant differences were determined by ANOVA test (*P* < 0.05).

To determine if DEF8 also works in organisms other than rice, we then ectopically overexpressed *DEF8* in *Arabidopsis* (Figure 7A). The results showed that when under the control condition, no significant difference was observed between the overexpression lines and the wild-type control, while when Cd was applied, root elongation was less affected in the overexpressing lines OE-1/OE-2 compared with the wild-type Col-0 (Figure 7, B and C). Moreover, Cd accumulation was higher in OE-1/OE-2 shoots than in Col-0, while lower accumulation was observed in roots (Figure 7D). These results suggest that DEF8 also works in *Arabidopsis* by promoting Cd mobilization from symplast to apoplast of the vasculatures, where the transpiration stream drives the mobilized Cd moving from roots to shoots. This finding indicates that DEF8 could be applied to modulate Cd accumulation in other plants.

## Discussion

In the past two decades, many genes involved in Cd uptake, translocation, or sequestration have been identified in the efforts to control Cd accumulation in rice grains. However, only very few of them have the ability to specifically regulate Cd translocation and accumulation, making it even harder to reduce Cd in grains without penalty in either plant growth or mineral homeostasis. In this study, we functionally characterized the defensin family member DEF8, which is highly expressed in rice grains, binds to Cd, and facilitates Cd efflux into apoplasts, thus we propose that DEF8 functions to unload Cd from phloem to grains, representing a possible mechanism and breeding marker to control grain Cd contents.

### DEF8 has dual function in either xylem loading or phloem unloading

DEF8 is a homolog sharing 60% similarity to the sequence of CAL1 in rice. Functional study revealed that both DEF8 and CAL1 bind to Cd and are secreted from symplast to apoplast likely in the form of protein:Cd complex, as DEF8 and CAL1 bind to Cd equally efficient at pH 7.5 and 5.5 (Figures 2 and 3; Luo et al., 2018), the values representing the symplast and apoplast acidity environment. In root parenchyma or pericycle cells, this secretion enables Cd loading into xylem vessels and hence promoting Cd long-distance transport from roots to shoots. Unlike CAL1, however, DEF8 also expresses in phloem cells in both root and grain vasculatures, and the same secretion pattern otherwise functions to remove Cd from phloem to apoplast, that is, unload Cd to grains when expressed in endosperm vascular bundle (Figures 3–6). The dual roles of DEF8 suggest that although highly similar to CAL1, it evolved new function due to the extra expression in phloem, which endows it to function in both xylem loading and phloem unloading, though the exact working mechanism remains to be further investigated. In terms of the functional role of DEF8 in rice root phloem, it might be unloading Cd in phloem to the adjacent xylem, thus preventing Cd recycling to roots and further promoting Cd root-to-shoot transport efficiency.

LCT1 was identified as a phloem Cd loading transporter (Uraguchi et al., 2011). A previous study proposed that OsHMA2 is also a phloem Cd loading transporter (Yamaji et al., 2013). However, to the best of our knowledge, a phloem Cd unloading transporter has not been identified yet. It was reported that more than 91% of grain Cd originates from phloem, and phloem Cd is preferentially bound to Cys and exists as a protein or thiol complex of about 13 KD (Tanaka et al., 2007; Kato et al., 2010). Given DEF8 is Cys-rich with a size of ∼6 KD, and its homolog CAL1 has been identified to present as dimers (Ochiai et al., 2020), we suspect that DEF8 might also dimerize to form a complex bound to Cd, and the complex is then translocated from phloem to apoplast via vesicle trafficking pathway (Figure 2J). From this aspect, although DEF8 is not a transporter, its binding and secretion function facilitates Cd efflux from phloem, thus unloading phloem Cd and representing a “secretion type” phloem Cd unloading mechanism.

Given DEF8 did not show the ability to translocate back to the symplast (Figure 2, D, F, and G), we postulate that the phloem DEF8:Cd unloaded to grains might be more likely from sources other than the root DEF8:Cd undergoing long distance transport to shoots. In other words, this complex might be formed in the spikelet phloem where DEF8 is synthesized on site but Cd is translocated from vegetative tissues via pathways including LCT1 or OsHMA2. Supporting evidence to our hypothesis came from a previous study, in which manipulation of CAL1 did not affect Cd accumulation in grains (Luo et al., 2018). From this aspect, the dual function for DEF8 might work separately to mediate Cd accumulation in vegetative and reproductive tissues. We note that the DEF8:Cd complex is theoretically proposed and has not been experimentally detected.

### Possible roles for DEF8 in processes other than mediating Cd distribution

One challenge in controlling grain Cd is how to specifically mediate Cd allocation without affecting other essential ions that are chemically similar to Cd. Most of the characterized key Cd transporters showed transport activity for Cd as well as for Fe, Zn, and Mn (Nakanishi et al., 2006; Ueno et al., 2010; Miyadate et al., 2011; Takahashi et al., 2011; Ishikawa et al., 2012; Sasaki et al., 2012; Yamaji et al., 2013; Cai et al., 2019; Pita-Barbosa et al., 2019; Chang et al., 2020), thus functional manipulation of these transporters inevitably result in unexpected side effect on nutrient metal homeostasis and plant growth or grain quality. In contrast, DEF8 is highly specific to Cd although it did show in vitro binding to Cu (Figure 3 and Supplemental Figure S2). Taking into account that DEF8 might work together with CAL1 to control the sequential processes in Cd loading to xylem and unloading from phloem to grains, we propose that genetic modification of both *DEF8* and *CAL1* could contribute additively to Cd allocation in rice without essential side-effect on either plant growth or metal homeostasis (Figure 6 and Supplemental Figures S3 and S4).

So why does DEF8 show such a high substrate specificity for Cd despite that Cd is not always present in soil? There is no solid clue to give any exact answer at present, but it is reasonable that DEF8 might have evolved other functions first, and later obtained metal binding activity loosely or closely relevant to its primary functions.

Given DEF8 is expressed at high level in rice grains and adjoint tissues (Figure 1C), and the expression keeps rising during grain irrigation and ripening (Supplemental Figure S1), we suspect that DEF8 might function in regulating seed dormancy and pre-harvest sprouting (PHS), a global issue with serious impact on crop quality and yield (Park et al., 2021; Sohn et al., 2021), for the following reasons: (1) abscisic acid (ABA) maintains seed dormancy and inhibits seed germination, while gibberellin (GA) breaks dormancy and promotes seed germination (Hartmann et al., 2011; Miao et al., 2018; Tuan et al., 2018, 2019). *DEF8* expression was strongly induced by ABA (Supplemental Figure S6), consistent with a proposed function of maintaining dormancy. (2) It was reported that defensins inhibited α-amylase (Lin et al., 2007), and GA promoted the synthesis of α-amylase during germination (Nakayama et al., 2002; Wang et al., 2018), thus it is reasonable that GA would inhibit *DEF8* expression (Supplemental Figure S6). (3) *DEF8* expression was highly up-regulated by drought stress and substantially inhibited by imbibition process (Supplemental Figure S6, A and B), providing further support to our hypothesis that DEF8 helps to keep dormancy. However, no significant germination phenotype was observed in either the *def8* mutants or the EE transgenic lines (Supplemental Figure S4, A and B). We suspect that germination under certain conditions may help, especially when considering that DEF8 shows binding activity to Cd and Cu.

Another possibility is that DEF8 might be involved in disease resistance. Previous studies suggest that defensins have a broad spectrum of antifungal activity (Thomma et al., 2002; Tantong et al., 2016; Weerawanich et al., 2018). CAL1, the homolog of DEF8, was also identified to have antifungal activity (Sagehashi et al., 2017; Ochiai et al., 2018, 2020). High level expression of *DEF8* in glume and rachis might help in preventing pathogen invasion. Moreover, Cu has been proposed to play a very important role in pathogen resistance (Yuan et al., 2010), and in vitro binding assay did indicate DEF8 bound to Cu (Supplemental Figure S2D). The consistency suggests that DEF8 possibly mediates disease resistance in combination with its role in Cu or even Cd binding. In terms of why we did not observe any apparent Cu accumulation phenotype in the *def8* mutants, it is possible that functional redundant genes to *DEF8* might exist in regulation of Cu homeostasis, or less likely that DEF8 might mediate Cu homeostasis in certain tissues, as was reported for the mystery of AtPCS2, which was finally identified to mediate Zn homeostasis in root tips (Tennstedt et al., 2009; Blum et al., 2010; Ding et al., 2013).

**Figure 8:**
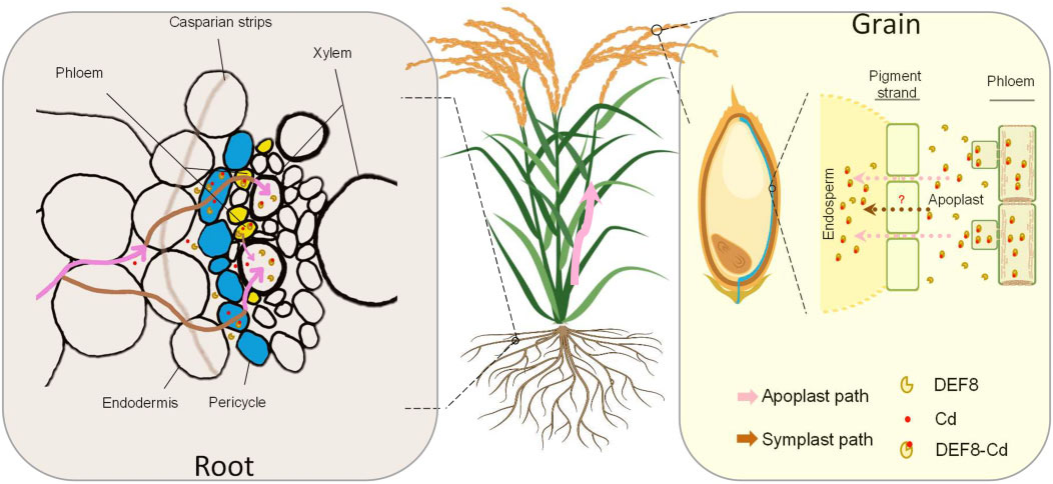
A working model for Cd allocation mediated by DEF8 in rice. We propose that DEF8 expressed in root pericycle and phloem cells facilitates Cd efflux into apoplast spaces by chelation and secretion, thus loading Cd into xylem for long distance transport from root to aerial parts, where Cd is further loaded into phloem, possibly via LCT1, HMA2, or other unidentified transporters. Grain Cd efflux from phloem to apoplast is facilitated by DEF8 via a similar mechanism in roots. The apoplast Cd possibly moves and accumulates in the endosperm via free diffusion or active transport across the membrane of the pigment strand cells surrounding the endosperm. The blue line in the grain indicates vascular bundles of brown rice. DEF8-Cd represents the complex which is not actually detected but by prospection from experimental evidence, meanwhile free DEF8 exists in the apoplast prior to Cd treatment.

In summary, we identified DEF8 as a dual role player in both xylem Cd loading and phloem Cd unloading, which chelates Cd and is secreted from symplast (regular cytosol or phloem) to apoplast compartments, thus driving Cd translocation from root to shoot and finally unloading Cd to rice grains (Figure 8). This Cd transport mechanism might be genetically modified to specifically control grain Cd level, though why this happens or if it is of any physiological importance remains an open question, especially when Cd pollution did not impose a serious concern until recently.

## Materials and methods

### Plant materials and growth conditions

Rice (*O. sativa*) genetic materials used in this study are in the ZH11 genetic background, and the *def8* mutants were generated by BIOGLE GeneTech (http://www.biogle.cn/). EE lines EE-9 and EE-16 were generated by transforming ZH11 with the construct *proDEF8::_f_DEF8-RFP/pCAMBIA1300*. Rice plants were grown in paddy fields with heavy metal pollution. Alternatively, uniformly germinated seeds were planted in 96-well plates without the bottoms and kept growing in Yoshida solution (pH 5.8) at 28°C, 60% relative humidity with a 13-h light/11-h dark photoperiod.

*Arabidopsis* transgenic lines OE-1 and OE-2 were generated by transforming the construct *35S::_f_DEF8-GFP/pCAMBIA1300* into Col-0 using the *Agrobacterium tumefaciens*-mediated floral dip method as described (Clough and Bent, 1998). *Arabidopsis* plants were grown at 21°C–23°C with 60% relative humidity and a 16-h light/8-h dark photoperiod.

### Phylogenetic tree construction and sequence alignment

Sequences of rice defensin proteins were downloaded from EMBL-EBI database and was aligned using neighbor-joining method of the ClustalX program. Phylogenetic tree was further constructed using MEGA7 and modified on the iTOL website (http://itol.embl.de/).

### Reverse transcription quantitative PCR

Total RNA was extracted using TRIzol reagent (Invitrogen). cDNAs were synthesized using oligo (dT) primers and PrimeScript Enzyme Mix I (TaKaRa). Quantitative PCR was performed (CFX Connect Real-Time System; Bio-Rad) using SYBR Premix Ex-Taq HS (TaKaRa) according to the manufacturer’s protocol. Primers used in these assays are listed in Supplemental Table S1, and expression data were normalized to *Actin1*.

### Protein purification and metal binding assay

Fragments of _f_DEF8 (full-length coding sequence) and _t_DEF8 (a truncated DEF8 without the SSP at amino acid sites between 1 and 33) were amplified by PCR (Supplemental Table S1) and cloned into the vector pCold-TF, respectively. The resulting constructs were further transformed into *E. coli* strain *BL21* (*DE3*). Recombinant proteins were induced by 0.2 mM isopropyl b-d-1-thiogalactopyranoside at 16°C for 20 h, and the cells were harvested and lysed using a French press. Cell debris was removed by centrifugation. The supernatant was purified using Ni-NTA agarose (Qiagen), and further eluted and concentrated. Metal binding assay was performed as described (Luo et al., 2018).

### Subcellular localization and protoplast isolation

The coding sequence of *_f_DEF8* was amplified using PCR primers (Supplemental Table S1), then fused with *mRFP* to generate the construct *35S::_f_DEF8-mRFP/PA7*, and the construct was transiently expressed in onion epidermal cells using a particle gun-mediated system (PDS-1000/He; Bio-Rad). The bombarded cells were held in the dark at 28°C for 16 h before confocal imaging. Additionally, the *proDEF8::_f_DEF8-mRFP*/*pCAMBIA1300* construct was transformed into ZH11, and indicated tissues of the transgenic lines were subjected to mRFP imaging through hand-sliced method. The mRFP fluorescence was excited at 561 nm line ray of the argon laser and observed between 572 and 610 nm with a gain set at 20 using a confocal laser scanning microscope (TCS-SP8; Leica).

For protoplast isolation, rice plants were grown to 2 weeks old in hydroponics supplemented with 5 µM Cd. Leaf sheaths from about 30 seedlings were cut into approximately 0.5 mm strips with propulsive force using sharp razors to isolate protoplasts as described (Luo et al., 2018). Metal concentration in protoplasts were determined by ICP-MS and normalized to corresponding total proteins.

### Western blotting

To test if DEF8 is subjected to long distance transport, proDEF8::_f_DEF8-mRFP was transformed into ZH11, then leaves, xylem sap, and guttation were sampled from the resulting transgenic plants at indicated stage. Total proteins were extracted from leaves using buffer E (125 mM Tris–HCl, pH 8.0, 1% [w/v] SDS, 10% [v/v] glycerol, and 50 mM NaS_2_O_5_). In total, 20 µg total proteins, 20 µL xylem sap, or 20 µL guttation was separated on 10% (w/v) SDS-PAGE gel and subjected to Western gel blotting according to the manufacturer’s standard protocol. Mouse anti-mRFP mAb (ABclonal, AE020; at 1:3,000 dilution) was used as primary antibodies. Horseradish peroxidase-labeled anti-mouse antibody (EpiZyme, LF101; at 1:5,000 dilution) was used as a secondary antibody. Membranes were visualized using an Immobilon Western Chemiluminescent HRP Substrate Kit (Sangong Biotech) and photographed with Image Quant LAS 400 mini.

### Histochemical localization

A 1,855-bp genomic fragment upstream of the *DEF8* start codon was amplified using primers listed in Supplemental Table S1. The fragments were verified by sequencing and cloned into the binary vector *pCAMBIA1300*. Putative transformants harboring *proDEF8::GUS*/*pCAMBIA1300* were selected in Yoshida solution containing 0.0025% (w/v) hygromycin B. GUS staining was performed with plants at indicated age. Samples were vacuum infiltrated until they sank to the bottom, then incubated for 12 h in GUS staining buffer containing 50 mM NaPO_4_, pH 7.4, 5 mM ferrocyanide, 5 mM ferricyanide, 0.05% (v/v) Triton X-100, 10 mM EDTA, and 1 mM X-Gluc (5-bromo-4-chloro-3-indolyl-b-d-glucuronide). Thin cross-sections (8 μm) were generated using a Leica RM 2165 microtome and imaged using the Nikon-SMA800.

### Quantification of ion content

Sampled tissues as indicated in the study were dried in 80°C oven for 2 days and digested in 1 mL 65% (w/v) HNO_3_ for at least 2 days at room temperature. Then the samples were boiled for 1–2 h until completely digested and diluted with 12 mL Milli-Q H_2_O. Ion contents were determined using ICP-MS as described.

### Statistical analyses

Details of statistical analyses are presented in the figure legends. Statistical analysis was performed on Prism software (GraphPad 8.40). The significance of the study was calculated using Student’s *t* test and one-way ANOVA test.

## Accession numbers

Sequence data from this article can be found in Rice Genome Annotation Project (https://rice.plantbiology.msu.edu/) or EMBL-EBI data libraries under accession numbers (see Supplemental Table S2).

## Supplemental data

The following materials are available in the online version of this article.

**Supplemental Figure S1.** *DEF8* expression is steadily elevated during rice ripening.

**Supplemental Figure S2.** Generation and characterization of rice genetic materials.

**Supplemental Figure S3.** Cd accumulation and uptake assay.

**Supplemental Figure S4.** Germination and Cd sensitivity assay.

**Supplemental Figure S5.** Important agronomic traits were not affected in the mutant and EE plants.

**Supplemental Figure S6.** *DEF8* expression under various environmental stimuli.

**Supplemental Table S1.** Primer sequences used in this study.

**Supplemental Table S2.** Accession numbers for genes used in this study.

## Supplementary Material

kiac423_Supplementary_Data

## Notes

J.-M.G. conceived and supervised the project. J.-M.G., T.-Y.G., and Z.-A.Q. designed the experiments. T.-Y.G. and Z.-A.Q. performed most of the experiments. S.-Y.C., J.Y., J.-M.W., and Z.-J.F. performed some of the experiments. J.-M.G., S.-Y.C., and T.-Y.G. wrote the manuscript.

The author responsible for distribution of materials integral to the findings presented in this article in accordance with the policy described in the Instructions for Authors (https://academic.oup.com/plphys/pages/general-instructions) is: Ji-Ming Gong (jmgong@cemps.ac.cn).
