# Supplementary material for: Dual-function DEFENSIN 8 mediates phloem cadmium unloading and accumulation in rice grains
Source: Plant Physiol. 2022 Sep 10;191(1):515–27. doi: 10.1093/plphys/kiac423 (PMC9806624; doi:10.1093/plphys/kiac423)
Supplement: kiac423_Supplementary_Data [file kiac423_supplementary_data.zip › kiac423_Supplementary_Data/Supplemental Data.pdf]

Supplemental Figure S1. ***DEF8*** **expression is steadily elevated during rice ripening**

A

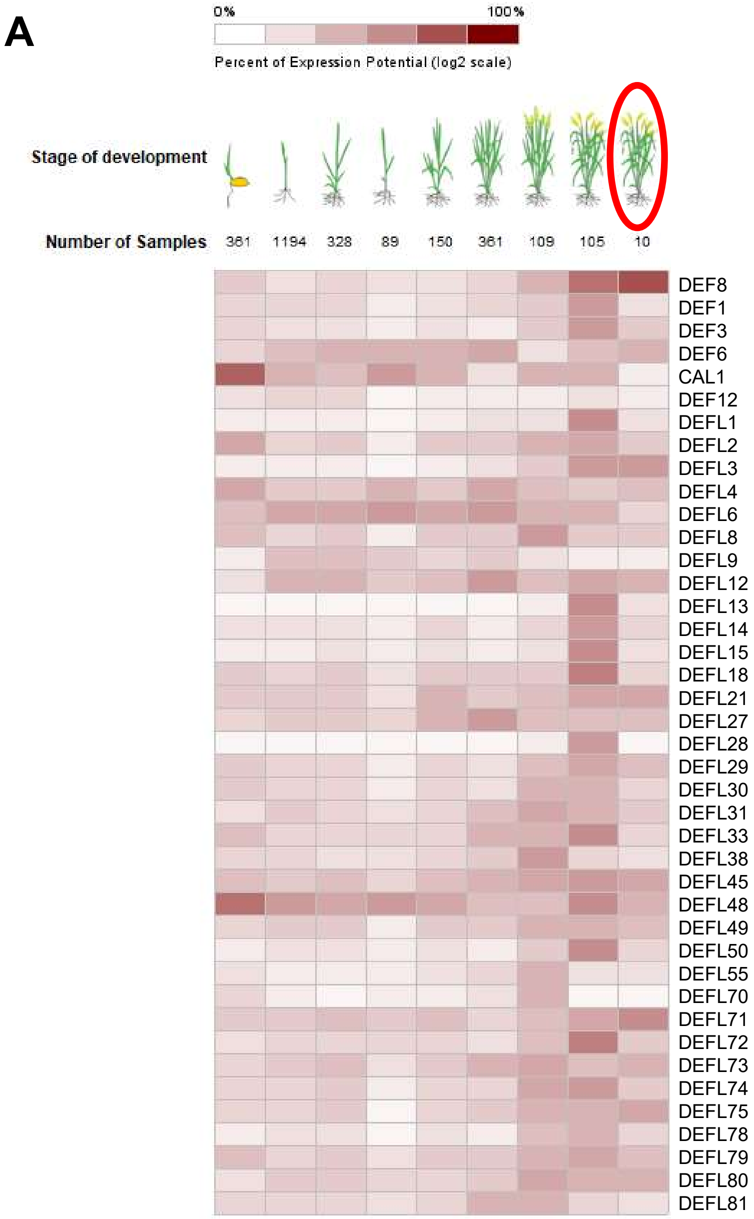

B

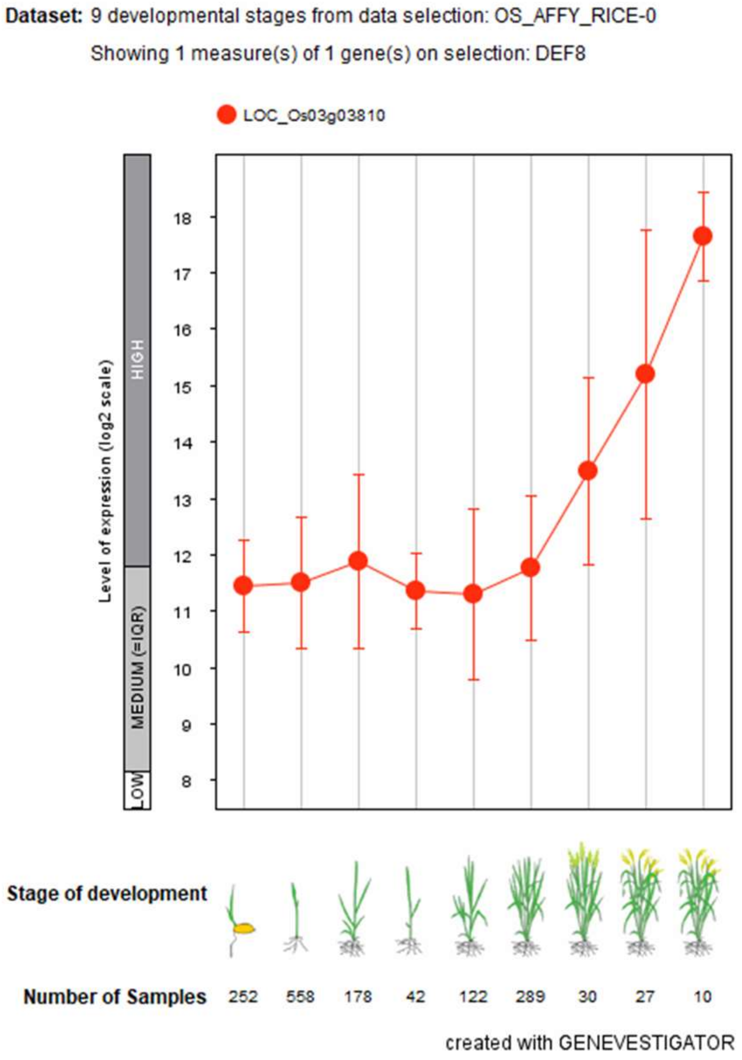

(A) Expression of the defensin family members at different developmental stages. Expression profiles were from Genevestigator Affymetrix platform. The darkest burgundy color represents the “maximum” level of expression for a given probe across all measurements available in the database.
(B) *DEF8* (Loc_Os03g03810) expression at different developmental stages as shown in Genevestigator. Value=mean ± SE. The sample size are showen in the numder of the samples.

Supplemental Figure S2. Generation and characterization of rice genetic materials

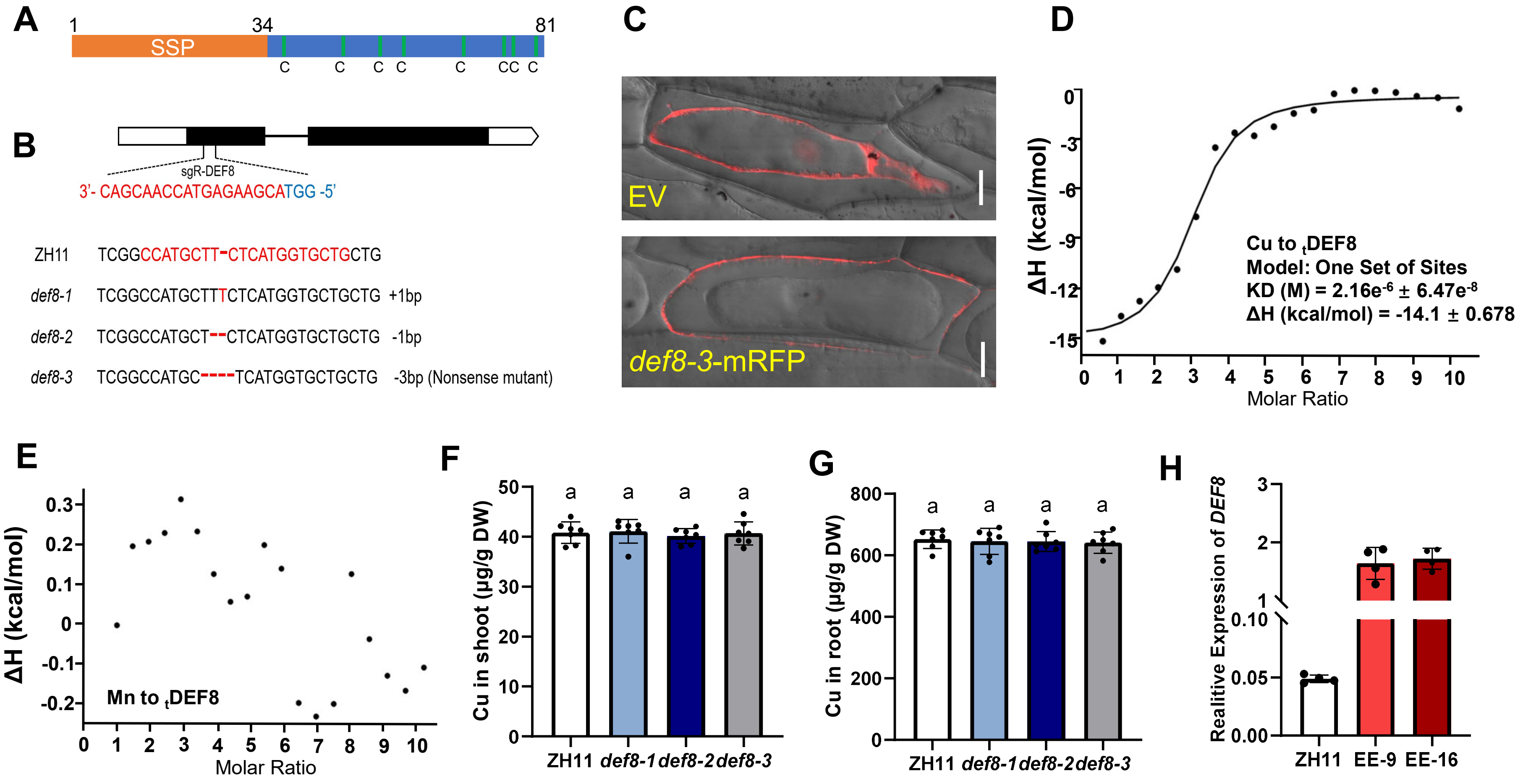

(A) Structural model of the DEF8 precursor with two parts. The first part indicated as orange box is the putative secretion signal peptide (SSP) predicted by web tool signalP 5.1 server. The second part represented by blue box is the mature protein sequence. Green bars indicate cysteine residues, and numbers indicate the sequence site of amino acids. **(B)** Identification of three *def8* mutant lines generated by CRISPR/Cas9. *def8*-1 has a single "T" insertion and *def8-2* has one nucleotide deletion in the coding region, while the *def8-3* mutant harbors a "TTC" deletion which resulted in loss of one amino acid in the secretion signal peptide region. **(C)** Onion epidermal cells transiently transformed with mRFP (EV) and *35S::$_f$def8-3-mRFP* ($_f$def8-3-mRFP) were incubated in 30% sucrose to induce plasmolysis and then imaged by confocal microscopy. Bar= 25 µm. **(D** and **E)** ITC analysis to determine binding of the recombinant protein $_t$DEF8 to Cu **(D)** and Mn **(E)** at pH 7.5. **(F** and **G)** Cu accumulation in mutant plants. 2 week old *def8* mutants were treated with 10 µM Cd for 7 days, then shoot **(F)** and root **(G)** were sampled for analysis. Values are mean ± SD, n = 7. Significant differences were determined by ANOVA test (*P* < 0.05). **(H)** *DEF8* expression in roots of EE-9 and EE-16. The transgenic plants were generated by transforming the construct *proDEF8::DEF8-mRFP/ pCAMBIA1300* into the wild type (ZH11). Values are mean ± SD, n = 4. *Actin1* was used as an internal control.

Supplemental Figure S3. Cd accumulation and uptake assay

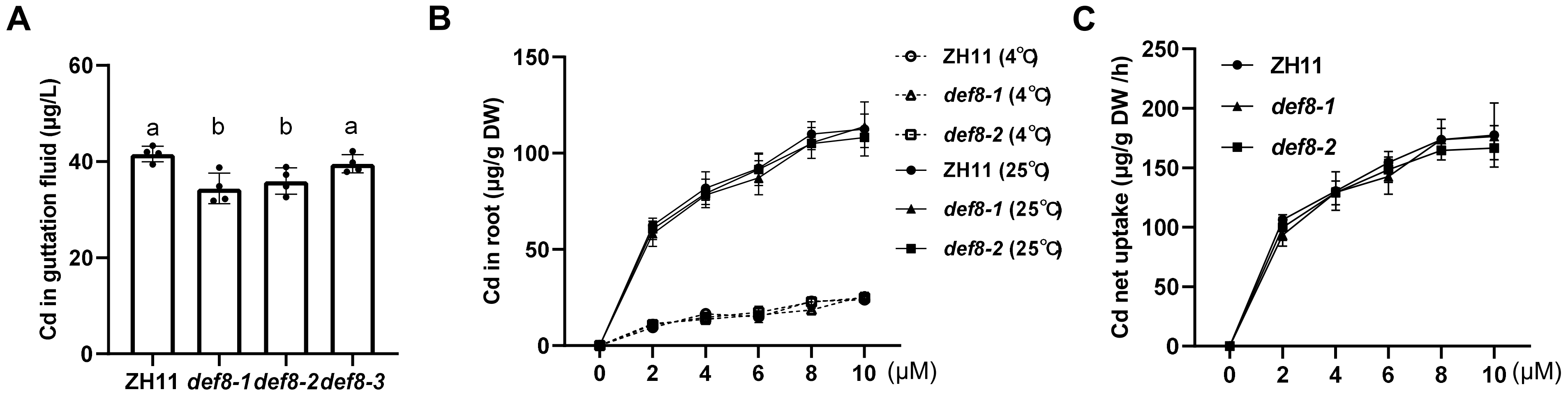

(**A**) Four days old seedlings were treated with 2 μM Cd for 1 day, then Cd contents in guttation fluid were determined for the wild type control (ZH11, *def8-3*) and the *def8* mutants. Values are mean ± SD, n = 4. Significant differences were determined by ANOVA test ($P < 0.05$). (**B** and **C**) Cd uptake in roots. Uptake assay was performed by exposing two week old seedlings of both the wild type (ZH11) and *def8* mutants to Cd of indicated concentrations at 25 and 4°C for 30 min (**B**). Net Cd uptake was calculated by subtracting the uptake at 4°C from that at 25°C (**C**). DW, dry weight. Data are mean ± SD, n = 3.

Supplemental Figure S4. Germination and Cd sensitivity assay

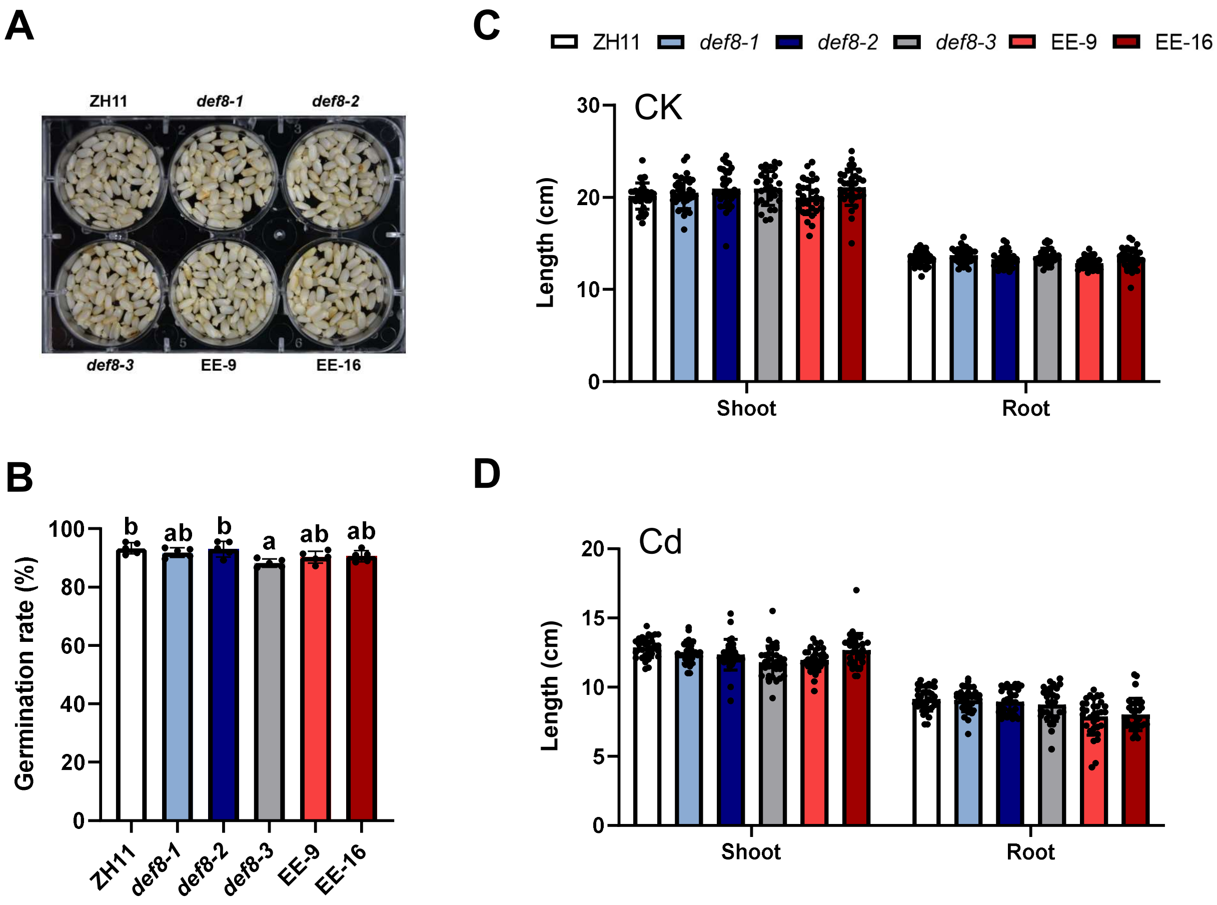

(A and **B)** Germination assay. Seeds of *def8* mutants, EE lines and their wild type were incubated in six-well plate for 2 days **(A)**, and germination rate was determined (B). Values are mean ± SD, n = 5. Significant differences were determined by Student's *t*-test (**P*<0.05).

(C and **D)** Cd sensitivity analysis. *def8* mutants, EE lines and their wild type were grown in hydroponics supplemented with 0 μM **(C)** or 5 μM Cd **(D)** for 2 weeks. Shoot and root length was then measured, and values are mean ± SD, n = 30-32.

Supplemental Figure S5. Important agronomic traits were not affected in the mutant and elevated expression plants

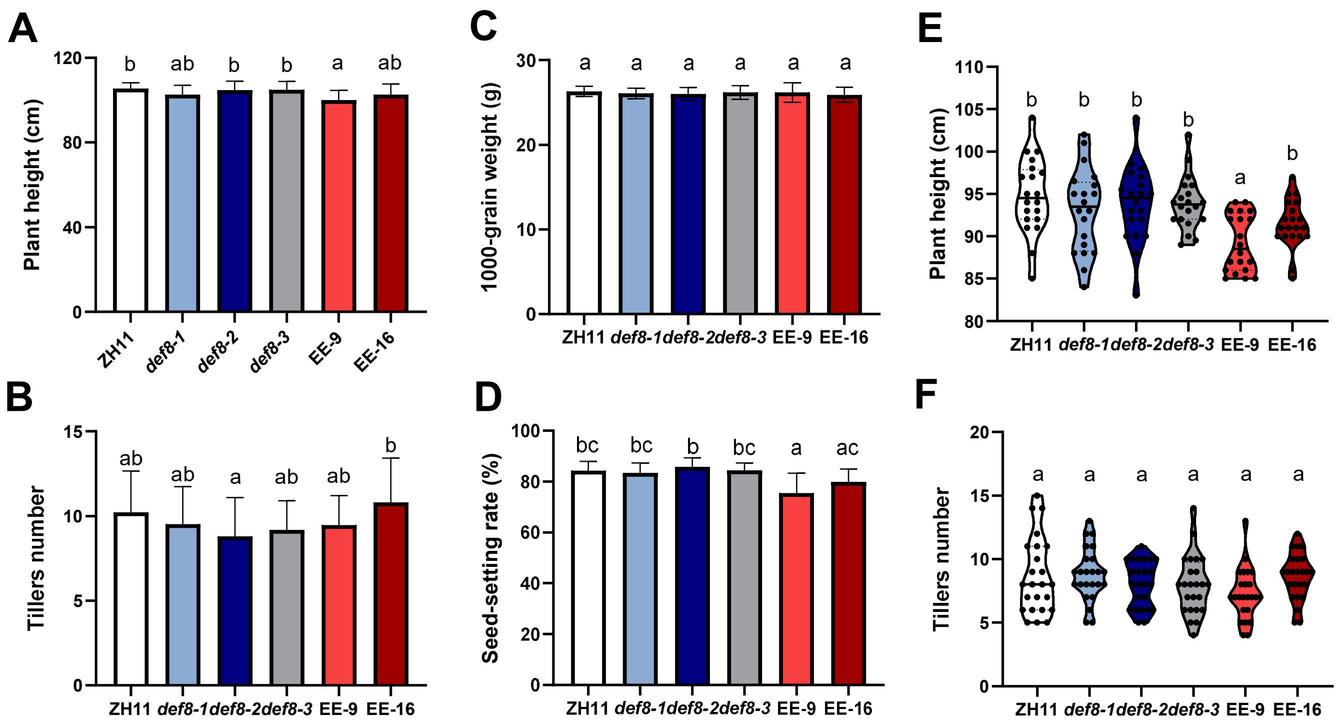

(A-D) Evaluation of important agronomic traits. Plant height **(A)**, tillers number **(B)**, thousand grain weight **(C)**, grain setting rate **(D)** were determined for *def8* lines (*def8-1*, *def8-2* and *def8-3*), EE lines (EE-9、EE-16) and their wild type control ZH11 grown in paddy field in Shanghai. Values are mean ± SD, n = 28 in **(A)**, 30 in **(B)**, and 16 in **(C** and **D)**. Significant differences were determined by ANOVA test ($P < 0.05$)

(E and **F)** Plant height **(E)** and tiller numbers **(F)** were determined for *def8* mutants, EE lines and the wild type control grown in Cd polluted paddy field in Zhejiang province. Values are mean ± SD, n = 20-24. Significant differences were determined by ANOVA test ($P < 0.05$)

Supplemental Figure S6. *DEF8* expression under various environmental stimuli

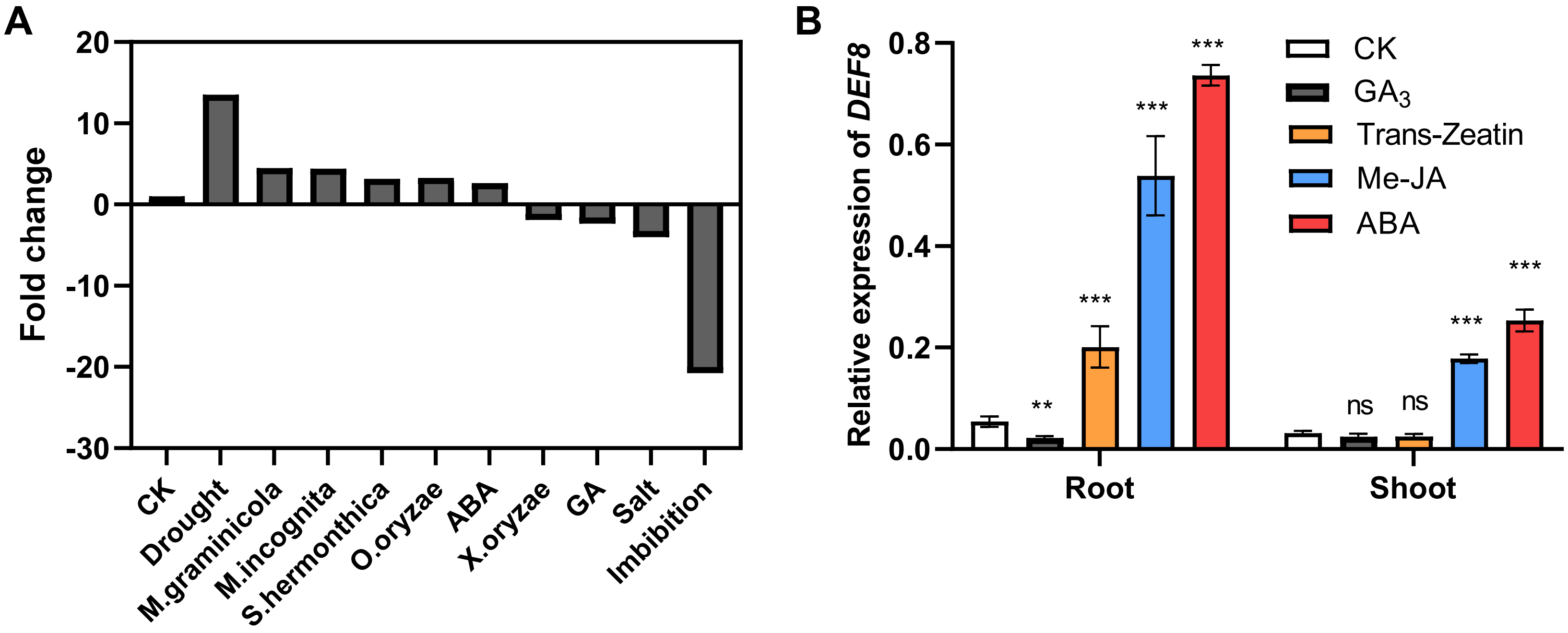

(A) Expression data for *DEF8* were obtained from the GENEVESTIGATOR (www.genevestigator.com). **(B)** RT-qPCR determination of *DEF8* expression. 2 week old seedlings were treated with 50 μM ABA, 50 μM Me-JA, 10 μM Trans-Zeatin or 10 μM GA_3 for 6 hours before sampling root and shoot tissues for RNA extraction. Values are mean ± SD, n=4 and normalized to *Actin1* and significancy was determined by Student's *t*-test. Asterisks (***P*<0.01, ****P*<0.001) indicate significant difference compared with the control (CK).

Supplemental Table S1. Primer sequences used in this study.

Assays	Destination	Forward primer 5'-3'	Reverse primer 5'-3'
Transient expres sion	*DEF8-mRFP/pA7*	TTCCTGCAGCCCGGG**GGATCC**ATGGAGGCTTCACGCAAGGT	CTCGGAGGAGGCCAT**ACTAGT**GGGGCAGGGCTTGGTGCACA
	$_{t}$*DEF8/pCold-TF*	CTCGGTACCCTCGAG**GGATCC**ATGCGGACGTGCGAGTCGCA	CAGGTCGACAAGCTT**GAATTC**TCAGGGGCAGGGCTTGGTGC
	$_{f}$*DEF8/pCold-TF*	CTCGGTACCCTCGAG**GGATCC**ATGGAGGCTTCACGCAAGGT	CAGGTCGACAAGCTT**GAATTC**TCAGGGGCAGGGCTTGGTGC
Plant transformation	*proDEF8-GUS/p1300 for Oryza sativa*	ACGACGGCCAGTGCC**AAGCTT**AGATGAAGAGAGGGGAAATG	GGACTGACCACCCGG**GGATCC**TTCTTCTTTCCCTTGGAACT
	fDEF8-mRFP/p1300 for Oryza sativa	ACGGGGGACTCTAGA**GGATCC**ATGGAGGCTTCACGCAAGGT	CTCGGAGGAGGCCAT**ACTAGT**GGGGCAGGGCTTGGTGCACA
	$_{f}$*DEF8-GFP/p1300 for Arabidopsis*	ACGGGGGACTCTAGA**GGATCC**ATGGAGGCTTCACGCAAGGT	GCCCTTGCTCACCAT**ACTAGT**GGGGCAGGGCTTGGTGCACA
qRT-PCR	$_{t}$*DEF8*	CAGAGCCACCGGTTCAAGG	GCAGGGCTTGGTGCACAT
	$_{f}$*DEF8*	GTATGCAACACGGAGGGCTT	TCGATCGACACGATGACACA
	Actin1	TCCATCTTGGCATCTCTCAG	GTACCCGCATCAGGCATCTG
Genotyping	identify *def8*	ATACGGTGATAAAGCAGCTG	GTGCAGTATTAATAGACTAG
